# Influence of Disturbance on Soil Respiration in Biologically Crusted Soil during the Dry Season

**DOI:** 10.1155/2013/408560

**Published:** 2013-12-18

**Authors:** Wei Feng, Yu-qing Zhang, Bin Wu, Tian-shan Zha, Xin Jia, Shu-gao Qin, Chen-xi Shao, Jia-bin Liu, Zong-rui Lai, Ke-yu Fa

**Affiliations:** Yanchi Research Station, College of Soil and Water Conservation, Beijing Forestry University, Beijing 100083, China

## Abstract

Soil respiration (Rs) is a major pathway for carbon cycling and is a complex process involving abiotic and biotic factors. Biological soil crusts (BSCs) are a key biotic component of desert ecosystems worldwide. In desert ecosystems, soils are protected from surface disturbance by BSCs, but it is unknown whether Rs is affected by disturbance of this crust layer. We measured Rs in three types of disturbed and undisturbed crusted soils (algae, lichen, and moss), as well as bare land from April to August, 2010, in Mu Us desert, northwest China. Rs was similar among undisturbed soils but increased significantly in disturbed moss and algae crusted soils. The variation of Rs in undisturbed and disturbed soil was related to soil bulk density. Disturbance also led to changes in soil organic carbon and fine particles contents, including declines of 60–70% in surface soil C and N, relative to predisturbance values. Once BSCs were disturbed, *Q*_10_ increased. Our findings indicate that a loss of BSCs cover will lead to greater soil C loss through respiration. Given these results, understanding the disturbance sensitivity impact on Rs could be helpful to modify soil management practices which promote carbon sequestration.

## 1. Introduction

Biological soil crusts (BSCs) are diminutive communities consisting of bacteria, cyanobacteria, fungi, lichens, mosses, and liverworts, all of which form a cohesive thin horizontal layer in association with the mineral soil surface [1, 2]. BSCs have several important identified roles in desert ecosystems, including significant contribution to carbon (C) and nitrogen (N) input (an estimate of 1.0 Pg ha^−1^ and 30 Tg ha^−1^ for net uptake of C and N by biocrusts in arid and semiarid regions), soil stability, and influence over patterns of erosion [3–5]. In total, the deserts of the world are estimated to contain 10 Pg C [6], with 56 × 10^12^ g C held in cyanobacterial biomass (a component of biocrusts) in arid and semiarid regions [7]. These numbers illustrate that BSC can sequester substantial C in the crust layer [8]. Soil stability is a primary control over carbon sequestration of managed ecosystems in arid and semiarid regions [9]. Most studies worldwide have shown that a protective cover of BSC can be a critical factor in soil stability [1, 3]. However, BSCs are highly susceptible to disturbance, especially in soils with low aggregate stability and dry conditions, such as sands in dry conditions [3, 10].

Studies on disturbance of BSCs have focused on soil nutrient losses, microbial communities, surface hydrology, erosion dynamic, and recovery rate [10, 11]. The impacts of disturbances on Rs in crusted soil have been less addressed. Rs is a major component of the biosphere's carbon (C) cycle and represents approximate three-quarters of total ecosystem respiration [12], and thus changes in Rs ultimately affect C storage. Therefore it is unclear whether disturbances on BSC will alter desert ecosystems from functioning as carbon sinks to functioning as carbon sources [13, 14]. A recent study in the Kalahari suggests a complex process because inhibition of BSC development led to greater Rs losses [15]. We argue that more work is needed to understand the disturbance of different types of crusted soil on Rs.

The landscape of the study area is characterized by a mosaic distribution of shrubs, BSCs, and bare land. Characteristics of the site are long dry periods and very infertile soils. Efforts to mitigate anthropogenic disturbance through fencing continue, but disturbance, such as human and livestock trampling, off-road driving, and annual grasses invasion, has increased. The objectives of the present study were to (1) compare Rs in undisturbed crusted soil with that in disturbed crusted soil of different types under drought conditions to allow estimate on changes in Rs following disturbance and (2) identify the controls which result in the difference of Rs between disturbed and undisturbed crusted soil.

## 2. Materials and Methods

### 2.1. Site Description

The research was conducted in the Yanchi Research Station (37°04′N~38°10′N, 106°30′E~107°41′E), at the south edge of the Mu Us desert, China. An elevation is 1550 m a.s.l. The prevailing climate is of the temperature arid and semiarid type, with average rainfall and temperature of 287 mm (62% of which fell between July and September) and 7.6°C, respectively. The soil has a bulk density of 1.61 g cm^−3^. All meteorological data were provided by the meteorological station of Yanchi County. Natural vegetation in the area is dominated by *Artemisia ordosica*. The soil surface between the shrubs is commonly covered by algae, lichen, and moss crusts which are mainly composed of *Microcoleus vaginatus*,* Oscillatoria chlorine*, *Collema tenax*, and *Bryum argenteum*. Species composition of BSCs were identified using the Opton West Germany 475200-9901 and Optex BK5000 microscopic technique and an extensive reference collection of field samples (Institute of Botany, Chinese Academy of Sciences, Beijing).

### 2.2. Experimental Design

Four most frequent soil cover types in *Artemisia ordosica* shrub were chosen, including algae crusted soil, lichen crusted soil, moss crusted soil, and bare land as control. For each cover type above, five 2 × 3 m plots at least 3 m apart were randomly selected as independent replicates. In each plot, two 3 m^2^ quadrats were established. One was scraped to 0% BSC cover (hereafter no moss, lichen, and algae crusted soils represented by NMC, NLC, and NAC, resp.); the other was undisturbed with intact BSCs (hereafter referred to as high moss crusted soils, high lichen, and high algae represented by HMC, HLC, and HAC, resp.). The reestablished BSCs within all disturbed crusted soil quadrats were carefully removed every two weeks after being scraped. The uppermost sediments at each plot were similar before disturbance because the geomorphology of the sites and chemical and textural properties of deeper (25 cm soil depth) soil samples were similar. In the bare land type, we selected five plots as independent replicates for Rs measurement. At each quadrat and plot in bare land, 8 cm tall, circular 80 cm^2^ PVC soil respiration collars were permanently inserted 5 cm into soils before one month of Rs measurement. Collars were placed >3 m apart from plants to minimize potential risk on Rs from roots and mycorrhiza.

During each measurement, we used aluminium containers with a surface area of 22.89 cm^2^ to collect soil samples. In each disturbed quadrat and bare land plot, samples were collected by inserting three 5.4 cm diameter cutting rings into soil about 5 cm deep, two were carefully removed to the aluminium containers to measure soil water content (SWC), soil organic carbon (SOC), total nitrogen content (TNC), and soil particle content, and the other cutting ring was put by a hole round cover with filter paper to examine soil bulk density (SBD). In each undisturbed soil quadrat, the BSC layer was carefully removed using a small shovel before sample being collected (as above) and then the removed BSC layer was carefully collected to examine SBD.

### 2.3. Field Respiration Measurements

Rs was measured with a non-steady-state flow-through chamber (LI-6400-09 soil chamber, volume 1000 cm^3^) connected to the portable Li-6400 (Li-COR, Lincoln, NE, USA). Measurements were performed at 2 h intervals on clear days from 7:00 a.m. to 19:00 p.m. on April 28, May 7, May 16, May 25, June 4, June 11, July 7, July 13, and July 25, 2010, during dry conditions and SWC was below 0.07 mm and photosynthesis of BSCs was limited (water compensation level of photosynthesis in BSCs is usually below 0.10 mm [16]). Soil temperature (Ts) of the top 2 cm of the soil profile was also recorded at each plot when conducting Rs measurements. The physical and chemical characteristics of the soils studied are shown in Table 1.

Description of thermally driven biological and soil microbial processes such as respiration is often based upon an Arrhenius model using the *Q*_10_ exponential relationship [17]:

(1) $$\begin{array}{c} \text{Rs}\left(T\right)=\text{R}{\text{s}}_{10}Q_{10}^{(T-T_{0})/10}, \end{array}$$

where Rs is the total soil respiration at temperature *T* and Rs_10_ the respiration at 10°C.

The sample was oven-dried at 105°C to determine SWC. The samples were air-dried and homogenized, then passed through a 2 mm sieve, and analyzed for SOC by potassium dichromate oxidation-outer heating, TNC by semimicro-Kjeldahl method, soil particle content by a laser particle analyzer (Mastersizer 2000, Malvern Instruments Ltd., Malvern, UK), and SBD of disturbed and bare land soil by a Soil Moisture Equipment (Santa Barbara, CA, USA) model 0200 soil core sampler. SBD and total porosity of BSCs layer were determined by the wax seal method [18] and SBD and total porosity were calculated using the following equations, respectively:

(2) $$\begin{array}{c} \gamma_{s}=\frac{100g_{1}}{\left\{\left[\left(g_{4}-g_{3}\right)/\rho_{1}-\left(g_{2}-g_{1}\right)/\rho_{2}\right]\times \left(100+W\right)\right\}}, \end{array}$$

where *γ*_*s*_ is soil bulk density (g·cm^−3^), same below; *g*_1_ is sample weight (g); *g*_2_ is sample weight completely wrapped by wax (g); *g*_3_ is original reading of electronic balance (g); *g*_4_ is reading of electronic balance with sample (g); *ρ*_1_ is specific gravity of water, *ρ*_1_ = 1.0 g·cm^−3^; *ρ*_2_ is specific gravity of wax, *ρ*_2_ = 0.9 g·cm^−3^; *W* is water content of sample:

(3) $$\begin{array}{c} f=\left(1-\frac{\gamma_{s}}{\rho_{s}}\right)\times 100, \end{array}$$

where *f* is soil total porosity (%); *ρ*_*s*_ is soil particle density (g·cm^−3^), *ρ*_*s*_ = 2.65 g·cm^−3^.

### 2.4. Statistical Analysis

Mean diurnal cycles of Rs and Ts were computed by averaging the hourly means for each time of day. Daily mean values were computed as the average of the hourly means. The daily mean values were used to examine the seasonal responses of Rs to Ts. Exponential equations (1) were used to simulate the relationships between Rs and Ts. To examine whether daily mean Rs and Ts differed between different cover types, we used repeated-measurement (RM: soil cover type and time) ANOVA. One-way ANOVA was used to test the effect of cover type on Ts for all tests, statistically significant differences were assigned to *P* values of <0.05. Prior to these analyses, data were tested for assumptions of normality and homogeneity of variances and were log-transformed when necessary. All the regression and ANOVA analyses were performed using the SPSS 15.0 statistical software (SPSS Inc., Chicago, IL, USA).

## 3. Results and Discussion

**Figure 1:**
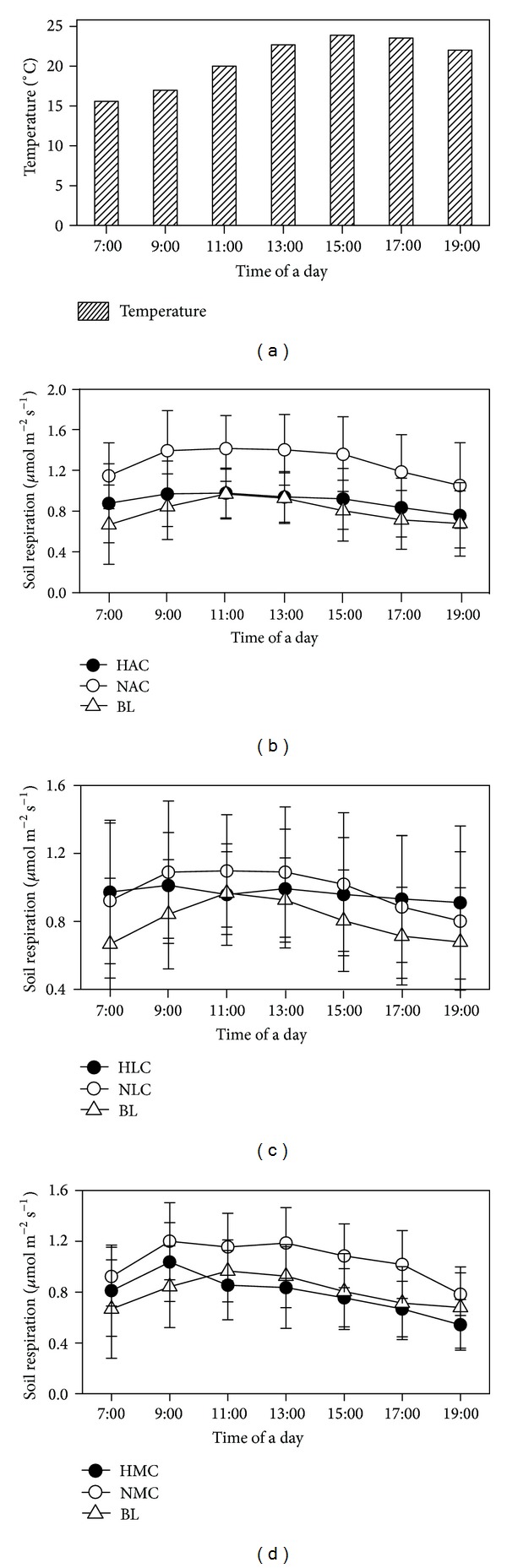
Diurnal variations in soil temperature (2 cm depth, bars a) and Rs in biocrusted soil (black solid circle), disturbed biocrusted soil (hollow circle), and bare land (upward triangle) in algae crusted soil (b), lichen crusted soil (c), and moss crusted soil (d). Error bars indicate standard deviation.

Rs showed signal peak curves at a day scale in four soil cover areas, with the highest values occurring in 13:00–15:00 (Figures 1(b), 1(c), and 1(d)). Rs was consistently higher in the NAC and NMC than that in HAC, HMC, and BL, respectively (Figures 1(b) and 1(d), *P* < 0.05). Temperature was not significantly different in the microsites of the three crusted soil cover areas and their mean values are those reported in Figure 1(a) (*P* > 0.05).

**Figure 2:**
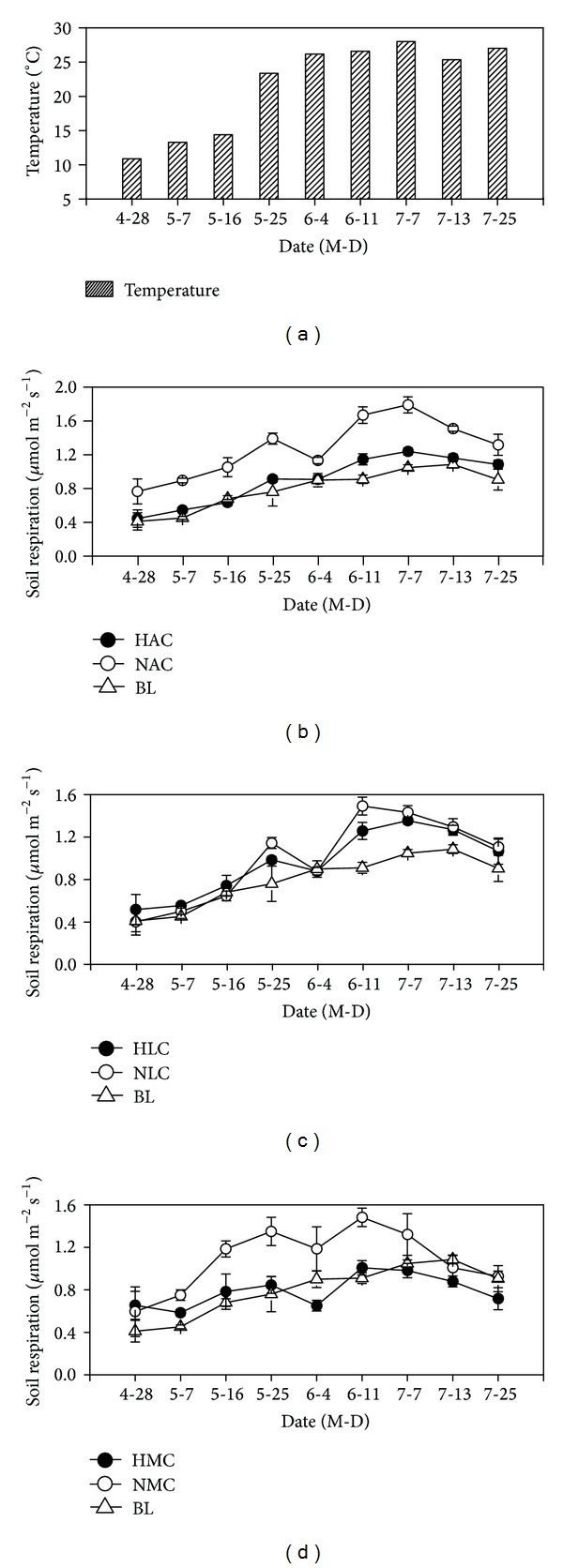
Mean daily soil temperature (a) and Rs in biocrusted soil (black solid circle), disturbance biocrusted soil (hollow circle), and bare land (upward triangle) in algae crusted soil (b), lichen crusted soil (c), and moss crusted soil (d). Error bars indicate standard deviation.

In the BL, daily mean Rs ranged from 0.41 ± 0.04 to 1.19 ± 0.73 *μ*mol m^−2^ s^−1^ (Figure 2(b)). In algae crust cover area, daily mean Rs ranged from 0.44 ± 0.11 to 1.24 ± 0.04 *μ*mol m^−2^ s^−1^ in the HAC and from 0.77 ± 0.14 to 1.79 ± 0.10 *μ*mol m^−2^ s^−1^ in the NAC. In lichen crust cover area, Rs ranged from 0.51 ± 0.14 to 1.35 ± 0.04 *μ*mol m^−2^ s^−1^ in the HLC and from 0.40 ± 0.13 to 1.49 ± 0.08 *μ*mol m^−2^ s^−1^ in NLC (Figure 2(c)). In moss crust cover area, Rs ranged from 0.58 ± 0.03 to 1.01 ± 0.07 *μ*mol m^−2^ s^−1^ in HMC and from 0.59 ± 0.23 to 1.48 ± 0.09 *μ*mol m^−2^ s^−1^ in NMC (Figure 2(d)). In algae and moss cover areas, RM ANOVA showed that daily mean Rs in NAC and NMC differed significantly higher than that in HAC, HMC, and BL, respectively (Table 2, *P* < 0.001). Interactions between microsites and measurement time had significant effects on Rs (Table 2, *P* < 0.001). In lichen crusted soil, RM ANOVA showed that daily mean Rs was not significantly different among HLC, NLC, and BL (Table 2, *P* > 0.05).

The Rs of NMC and NAC was significantly (*P* < 0.001) higher than that of HMC and HAC, respectively, which is consistent with the result studied in Kalahari [15]. Soil fertility could not be explanations for the result, as the soil fertility (SOC and TNC) in 0–5 cm under BSC layer in undisturbed soils was significantly higher than that in 0–5 cm in the disturbed soils (Table 1). The effect of SBD offsets that of soil fertility. The result is mainly due to SBD (*P* < 0.001) (Table 1). Similar observations were also reported by Novara et al. [19] and Pengthamkeerati et al. [20] who found a significant negative correlation of SBD with Rs. This occurs as increases in SBD reduce gas diffusivity which is linked with oxidation rate and consequently rates of soil respiration and CO_2_ emission [21].

There were no differences of carbon release observed in HMC, HLC, HAC, and BL under dry conditions, regardless of the strong difference in organism. This result indicated that respiration of the organisms is not the main driver factor under dry condition. Consistent with our study, the same difference was observed between the two crusted (moss and cyanobacteria/lichen crusted soils) soils and bare land in the Gurbantünggüt desert, north China [22], and no significant difference in carbon release between biocrust microsites in the Iberian Peninsula [23].

In disturbed and undisturbed crusted soil and bare land, daily mean Rs fluctuated exponentially with Ts at seasonal scale (HAC: *R* ^2^ = 0.94, *Q*_10_ = 1.45, *P* < 0.01; NAC: *R* ^2^ = 0.78, *Q*_10_ = 1.67, *P* < 0.01; BL: *R* ^2^ = 0.78, *Q*_10_ = 1.38, *P* < 0.01; HLC: *R* ^2^ = 0.86, *Q*_10_ = 1.65, *P* < 0.01; NLC: *R* ^2^ = 0.87, *Q*_10_ = 1.84, *P* < 0.01; HMC: *R* ^2^ = 0.42, *Q*_10_ = 1.16, *P* < 0.01; NMC: *R* ^2^ = 0.50, *Q*_10_ = 1.34, *P* < 0.01) (Figures 2(b), 2(c), and 2(d)). The average *Q*_10_ value in our study is lower than the *Q*_10_ value found in other ecosystems [23, 24]. However, our results are consistent with those of Fernandez et al. [25] and Thomas et al. [15] in dry season, as they found that low soil moisture limited *Q*_10_. However, the *Q*_10_ values were more sensitive in the disturbed soils than those in the undisturbed soils. The specific reason for the difference, however, is unclear. It may be caused by a difference in organisms and carbon fraction (labile and resistant) or by SOC quality variation in the disturbed and undisturbed crusted soil [26, 27], which need further investigation to understand.

Without protective cover from BSC, wind preferentially removes fine-grained material relative to coarse-grained material (Table 1), which appears to be an important mechanism leading to nutrient depletion in disturbed sites [28]. Disruption to the BSCs also adversely affects the composition and metabolic activity of the autotrophic organisms and their ability to fix CO_2_ and N_2_ [15]. The findings indicate crusted soils can rapidly become a net carbon source when they were disturbed in dry condition. This study provides significant insights that management should consider in long dry period deserts to keep BSC intact for preventing the greater loss of carbon from soil.

## 4. Conclusion

In conclusion, disturbance of BSCs will potentially lead to greater *C* losses in gases respired and *C* input in soils under dry condition. The results suggest that crusted soils protect strictly to sequester CO_2_ and can rapidly become a net source of CO_2_ if disturbed in dry condition. Therefore, disturbance of BSCs soil should be a consideration for management related to the long-term sustainability in dry-land environments.

## Figures and Tables

**Table 1:** Physical and chemical characteristics of the study sites. Values are the mean ± standard error.

Crust type		SOC (%)	TNC (%)	SBD (g·cm^−3^)	TP (%)	Particle content (<0.05 mm) (%)
Algae crust	HAC	0.34 ± 0.13	0.02 ± 0.01	1.69 ± 0.10	36.98 ± 1.40	6.16 ± 1.14
	NAC	0.28 ± 0.17	0.01 ± 0.01	1.50 ± 0.10	42.26 ± 5.57	5.67 ± 2.25
Lichen crust	HLC	1.33 ± 0.09	0.07 ± 0.01	1.60 ± 0.03	39.62 ± 1.10	8.43 ± 1.41
	NLC	0.67 ± 0.12	0.05 ± 0.01	1.49 ± 0.08	43.49 ± 1.89	7.00 ± 0.35
Moss crust	HMC	2.14 ± 0.19	0.10 ± 0.02	1.70 ± 0.45	35.84 ± 1.60	11.07 ± 0.81
	NMC	1.07 ± 0.12	0.09 ± 0.01	1.20 ± 0.01	54.72 ± 0.52	8.37 ± 1.71
Bare land		0.21 ± 0.14	0.02 ± 0.01	1.61 ± 0.07	39.24 ± 5.54	8.74 ± 0.21

SOC: soil organic carbon; TNC: total nitrogen content; TP: total porosity. HMC, HLC, and HAC, respectively, represent high moss crusted soils, high lichen, and high algae crusted soil. NMC, NLC, and NAC, respectively, represent no moss, lichen, and algae crusted soils; SBD is soil bulk density.

**Table 2:** The effects of soil cover type (*C*), measurement time (*T*), and their interactions (*C* × *T*) on mean daily Rs (*μ*mol m^−2^ s^−1^) in the three biocrusted soil cover areas.

	Algae crust cover area			Lichen crust cover area			Moss crust cover area		
	df	F	P	df	F	*P*	df	F	P
*C*	2	33.65	0.046	2	8.33	0.052	2	37.39	<0.010
*T*	8	39.27	<0.001	8	201.70	<0.001	8	26.94	<0.001
*C* × *T*	16	5.59	<0.001	16	4.62	<0.05	16	4.34	<0.050

df is degree of freedom; *F*: *F*-test; *P* is the significance level at <0.05.
